# Radiological extranodal extension in head and neck cancers: current evidence and challenges in imaging detection and prognostic impact

**DOI:** 10.1093/bjro/tzaf021

**Published:** 2025-08-26

**Authors:** Nivedita Chakrabarty, Abhishek Mahajan

**Affiliations:** Department of Radiodiagnosis, Tata Memorial Centre, Advanced Centre for Treatment, Research and Education in Cancer (ACTREC), Homi Bhabha National Institute (HBNI), Mumbai, Maharashtra, 400012, India; Department of Imaging, The Clatterbridge Cancer Centre NHS Foundation Trust, Liverpool, L7 8YA, United Kingdom; Faculty of Health and Life Sciences, University of Liverpool, Liverpool, L7 8TX , United Kingdom

**Keywords:** radiological extranodal extension, head and neck cancers, adverse prognostic indicator, 9th version AJCC staging

## Abstract

Extranodal extension (ENE) is an established adverse prognostic indicator for head and neck cancers (HNC), and its presence entails adjuvant chemoradiotherapy, hence, it had been incorporated for the first time as the advanced regional node N3b category in the 8th edition of the Union for International Cancer Control (UICC)/American Joint Committee on Cancer (AJCC) Tumour Node Metastasis (TNM) classification for cancers of the oral cavity, human papillomavirus-negative oropharynx, hypopharynx, larynx and major salivary gland carcinomas. Pathological ENE is available for cases which are operated on, but cases which are managed non-surgically or unfit for surgery rely on imaging for providing the information on ENE, and this has prompted researchers across the globe to devise radiological grading for ENE. Radiological ENE has finally been given due credit and incorporated in the 9th version of AJCC TNM staging for nasopharyngeal carcinoma, which came into effect from January 2025. Knowledge of ENE status on baseline imaging prior to operation also helps in counselling patients regarding prognosis and planning adjuvant treatment. In this article, we have comprehensively reviewed the radiological/imaging ENE (rENE/iENE) grading proposed by researchers worldwide, extensively reviewed the existing evidence and challenges of using rENE/iENE for staging, grading, prognosticating and treating HNC, and also discussed the future scope of using rENE/iENE for managing patients with HNC of all the subsites, including thyroid cancers. Artificial intelligence-based studies for predicting rENE/iENE have also been discussed briefly.

## Introduction

Extranodal extension (ENE) refers to the process of extension of a tumour from within the metastatic lymph node into the perinodal tissue through disruption and perforation of the lymph nodal capsule. Various studies have shown the presence of ENE to be an adverse prognostic indicator irrespective of the site of origin of cancer.^1–20^ Head and neck cancers (HNC), including head and neck squamous cell carcinomas (HNSCC), nasopharyngeal and thyroid cancers, have been shown to be associated with increased risk of distant metastasis, poor locoregional control, decreased overall survival, and increased risk of recurrence in the presence of ENE.^2^^,^^21–28^ The 8th edition of the Union for International Cancer Control (UICC)/American Joint Committee on Cancer (AJCC) had incorporated ENE in the advanced regional node N3b category of the Tumour Node Metastasis (TNM) classification for cancers of the oral cavity, human papillomavirus (HPV)-negative oropharynx, hypopharynx, larynx and major salivary gland, but not for nasopharyngeal and HPV-positive oropharyngeal carcinomas.^29–31^ Most of the initial studies focused on pathological ENE, however, not all the HNC are operated on. The need for prognosticating inoperable oral cancers, laryngeal cancers requiring organ preservation (based on the Veterans Affairs Laryngeal Study Group by Wolf et al^32^) oropharyngeal and hypopharyngeal cancers which are not operated on (based on the study by Lefebvre et al^33^) and nasopharyngeal cancers, in which radiotherapy is the prime treatment modality, necessitated the development of imaging-based criteria for detecting ENE. Radiological/imaging ENE (rENE/iENE) has finally been given due credit and incorporated in the recent 9th version of AJCC TNM staging for nasopharyngeal carcinoma (NPC), which came into effect from January 2025.^34^ Researchers across the world have tried to ascertain the diagnostic accuracy and prognostic value of radiological/imaging ENE (rENE/iENE) in HNSCC, some have attempted to grade ENE, while others have worked on artificial intelligence-based methods for predicting ENE on imaging. Most of the researchers have used ‘rENE’ to denote radiological ENE, hence, we have followed the same in our article. Though rENE has emerged as a strong prognostic factor in HNC, standardised criteria and a grading system for image-based prognostication are still lacking.

In this article, we have comprehensively reviewed rENE grading proposed by researchers worldwide and have extensively reviewed the existing evidence and challenges of using rENE for staging, grading, prognosticating and treating both viral-related (HPV or Epstein-Barr Virus) and unrelated HNC. We have also discussed the future scope of using rENE for managing patients with HNC of all the subsites, including thyroid cancers.

## Pathological ENE

Histopathology remains the gold standard for ascertaining the presence of ENE but is not without limitations. Histopathologically, ENE is defined as the extension of a tumour from the lymph node through the fibrous capsule into the surrounding tissues.^35^ Incomplete lymph node capsule, due to sampling error or thinned-out capsule, continuity of the primary tumour with the node, and confluent lymph nodes are the areas where ambiguity may exist regarding histopathological assessment of ENE.^35^^,^^36^ Prognostic impact of pathological ENE (pENE) was first observed by Johnson et al in cervical nodes.^37^ Various studies have been undertaken since then demonstrating poor survival outcomes in HNSCC with pENE.^38–42^

**Figure 1.**
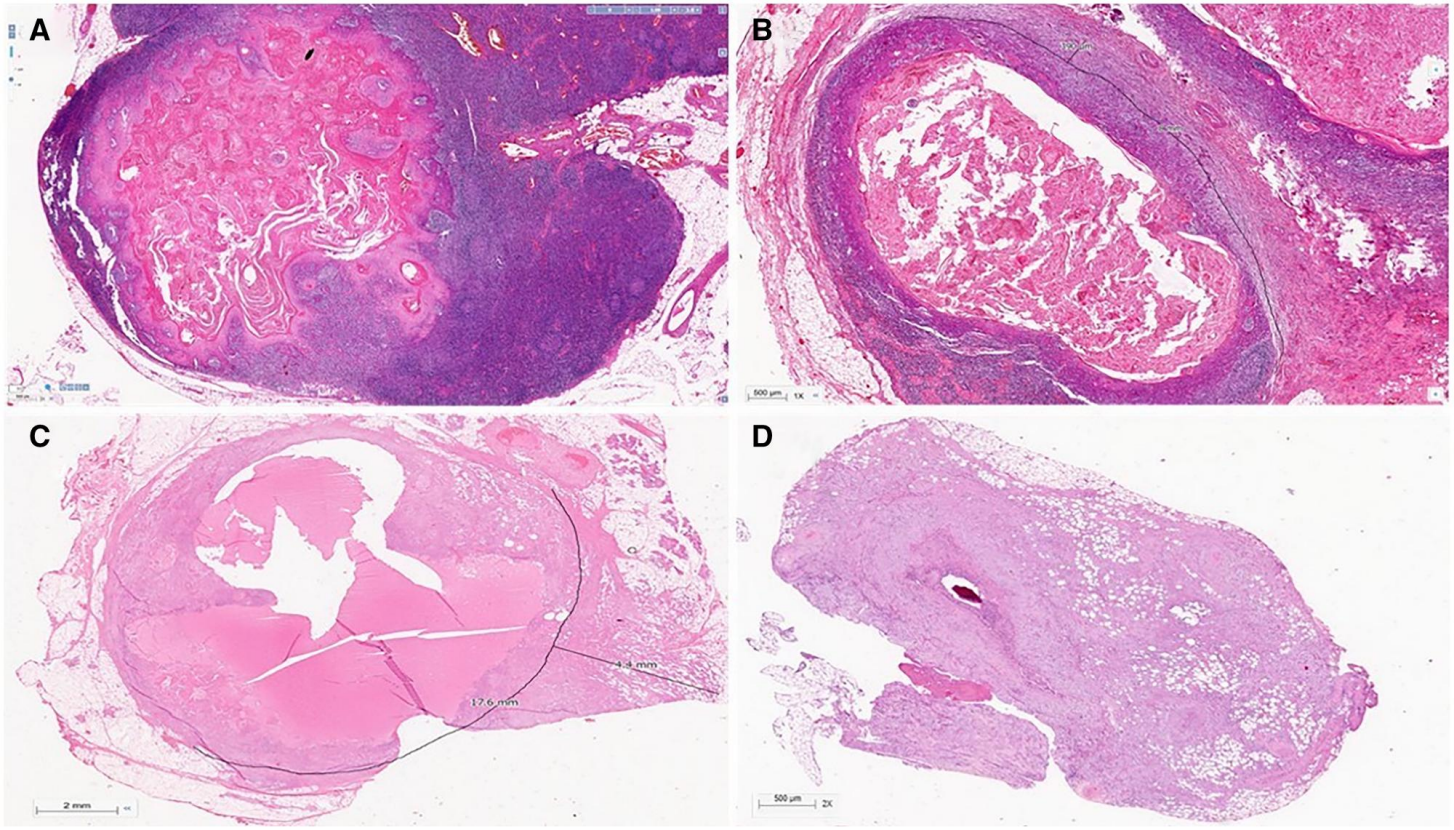
Involved node with and without pathological extranodal extension and soft tissue deposit. (A) Low magnificent Hematoxylin and Eosin (HE) stained section of an involved node shows absence of ENE (tumour does not extend beyond the node capsule). (B and C) Involved nodes with tumour deposits showing minor (≤2mm in largest extent) [b] and major ENE (>2mm in largest extent) [c], respectively, from an imaginary line that traces the contour of the node capsule. (D) Soft tissue deposits (as shown) are tumour deposits in the neck where no nodal architecture/shape is discernible.

pENE has been broadly divided into macroscopic/major (> 2 mm extent) with gross soft tissue involvement and microscopic/minor (≤ 2 mm extent), which is visible histopathologically only.^5^^,^^35^^,^^36^ Soft tissue deposits are metastases without any discernible nodal architecture.^43^  Figure 1 shows histopathologic images of involved nodes without ENE (Figure 1A), with minor (Figure 1B) and major ENE (Figure 1C), and with soft tissue deposit (Figure 1D).

Wreesmann et al found a cut-off of 1.7 mm for HNSCC, and Mamic et al found a cut-off of 1.9 mm for metastatic oral cavity SCC (OCSCC) for ENE to be prognostically significant for survival outcome, in keeping with the recommendation cut-off of 2 mm proposed by the AJCC.^38^^,^^39^ On the contrary, a study done by Tirelli et al in non-HPV-related HNSCC demonstrated a 3-year overall survival of 46% in the ENE minor group and 38.9% in the ENE major group, with no statistically significant difference between the two groups.^40^ A recent study has shown that major ENE is an independent prognostic factor in HPV-positive oropharyngeal squamous cell carcinoma (OPSCC) as well.^36^ Despite being an established poor prognostic factor, consensus on standardised lymph node sampling as well as reporting of pENE is yet to be achieved.

## Diagnostic accuracy and classification/grading of radiological ENE

Contrast-enhanced magnetic resonance imaging (CEMRI) is the baseline imaging modality of choice for evaluation of oropharyngeal squamous cell carcinoma (OPSCC), nasopharyngeal cancer (NPC), and tongue carcinoma, whereas contrast-enhanced computed tomography (CECT) is the modality of choice for evaluating gingivobuccal cancers and hypopharynx. CECT and CEMRI are complementary for evaluation of larynx. Same cross-sectional imaging is used for evaluation of both primary and neck nodes, with the additional role of ultrasound (US) for neck nodes and thyroid.^44–51^

The need for developing imaging-based criteria for ENE prompted many researchers across the globe to evaluate the diagnostic accuracy of various imaging modalities for assessing ENE, using histopathology as the gold standard, as shown in Table 1.^52–73^ Url et al and Prabhu et al concluded that CT scans had a high specificity for diagnosing ENE, and Almulla et al found CT and MRI to have a similar specificity of 95% for diagnosing ENE.^52^^,^^56^^,^^66^ King et al found similar performance of CT and MRI for predicting pENE with a diagnostic accuracy of 73% and 80% and a specificity of 93% and 86%, respectively.^67^ Both Aiken et al and Randall et al compared the diagnostic accuracy of CT with histopathology and concluded that presence of necrosis on a preoperative CT scan strongly correlated with pathological ENE.^55^^,^^59^ Kim et al found that along with the shape of the node, the presence of more than four metastatic nodes and the location of metastatic nodes to lower cervical region increased the predictive power of baseline CT for pathological ENE in oropharyngeal squamous cell carcinoma (OPSCC).^60^ Abdel-Halim et al and Su et al in their systematic review and meta-analysis, compared the diagnostic performance of CT, MRI, US and positron emission tomography (PET) CT with histopathology. Abdel-Halim et al concluded that PET CT had a significantly higher sensitivity of 80% than CT (76%) and MRI (72%) for diagnosing ENE in HNSCC, whereas Su et al found CT to have the lowest sensitivity, with similar specificity for all the imaging modalities.^72^^,^^73^ In the systematic review and diagnostic meta-analysis conducted by Park et al, the pooled sensitivity and specificity of CT and MRI were 73% and 83% and 60% and 96%, respectively for HNSCC.^69^ Park et al found central node necrosis to have a significantly higher pooled sensitivity (81%) and infiltration of adjacent planes to have a significantly higher pooled specificity (94%).^69^

**Table 1.** Diagnostic accuracy of imaging modalities for detecting extranodal extension (ENE) using pathological ENE as the gold standard for head and neck squamous cell carcinoma.

Authors	Type of study	Nature of study	Sample size	Subsite	Outcome	Comments
**Imaging modality-CT**						
Url et al^52^	Diagnostic	Retrospective	49	No specific HNSCC subsite	S=73% Sp=91%	CT has high specificity for ENE detection
Faraji et al^53^	Diagnostic	Retrospective	73	HPV+ OPSCC	For irregular nodal margins: S=28%-45% Sp=94%-95% PPV=80%-82% NPV=64%-73% For absence of perinodal fat plane: S=87%-96% Sp=34%-50% PPV=53%-59% NPV=62-63%	Absence of perinodal fat plane and presence of irregular nodal margins were the most sensitive and specific features, respectively for identifying ENE.
Maxwell et al^54^	Diagnostic	Retrospective	65	p16+ HNSCC	S=47%-55% Sp=70%-85% A=62%-63% PPV=72%-82% NPV=53%	CT not an accurate method for reliably determining the presence of ENE in p16-positive HNSCC patients.
Aiken et al^55^	Diagnostic	Retrospective	111	OCSCC	S=68% Sp=88%	Presence of necrosis was the best radiologic predictor of pathologically proven ENE
Prabhu et al^56^	Diagnostic	Retrospective	432	OCSCC and laryngeal cancer	S=43.7% Sp=97.7% PPV=82.6% NPV=87.3%	CT criteria of adjacent structure invasion was a better predictor for pathological ENE than irregular borders/fat stranding.
Carlton et al^57^	Diagnostic	Retrospective	93	No specific HNSCC subsite	S=57-66% Sp=76%-81% A=67%-70% PPV=80%-82%	Interobserver agreement was highest for central necrosis
Noor et al^58^	Diagnostic		80	p16+ OPSCC	S=56.5%-73.3% Sp=60.9%-66.7%	Presence of perinodal fat stranding significantly associated with pENE
Randall et al^59^	Diagnostic	Prospective	40	OCSCC	S=91% NPV=88%	Central node necrosis on preoperative CT scans strongly associated with pENE
Kim et al^60^	Diagnostic	Retrospective	108 (total OPSCC, out of which 76 were HPV +)	OPSCC	All OPSCC Nodal margin-related related feature: S=86.6% Sp=63.4% Nodal burden-related feature: S=56.7% Sp=90.2% Both features: S=53.7% Sp=92.7% HPV+ OPSCC Nodal margin-related related feature: S=89.1% Sp=63.3% Nodal burden-related feature: S=56.5% Sp=86.7% Both features: S=54.3% Sp=90%	Imaging parameters for nodal margin breakdown (indistinct capsular contour, irregular margin, and perinodal fat stranding) and nodal burden (nodal matting, lower neck metastasis, and presence of >4 lymph node metastases) were significant predictors of pENE.
Souter et al^61^	Diagnostic	Retrospective	149	No specific HNSCC subsite	S= 66%-80% Sp= 90%-91% PPV=85%-87%	
Carvalho et al^62^	Diagnostic	Retrospective	28	No specific HNSCC subsite	S= 62.5% Sp= 60% A=62%	CT may not detect 37.5% of cases with ENE
Chai et al^63^	Diagnostic	Retrospective	100	No specific HNSCC subsite	S= 49%-65% Sp=54%-84% A=61%-62% PPV=71%-84% NPV=48%-49%	CT is a poor predictor of pathological ENE
Geltzeiler et al^64^	Diagnostic	Prospective	100	HPV related OPSCC	S=55% Sp=94% PPV=91%	Diagnostic performance for ≥ 3 suspicious nodes on CT has a high predictivity for pENE
Patel et al^65^	Diagnostic	Retrospective	27	HPV related OPSCC	Prediction of pENE > 2mm S=88%-100% Sp=52.6%-63.2% PPV=43.8%-53.3% NPV=90.9-100%	Low PPV for HPV positive OPSCC
**Imaging modalities- CT and MRI**						
Almulla et al^66^	Diagnostic and prognostic	Retrospective	483 (307 CT and 176 MRI)	OCSCC	For CT: A=80% S=61% NPV=74% Sp=95% For MRI: A=63% S=40% NPV=53% Sp=95% 3-year OS: Significantly reduced OS (31%) for rENE.	CT showed improved S, NPV and A but similar Sp compared to MRI.
King et al^67^	Diagnostic	Prospective	17	No specific HNSCC subsite	For CT: A=73% S=65% Sp=93% PPV=96% NPV=50% For MRI: A=80% S=78% Sp=86% PPV=94% NPV=60%	No significant difference between CT and MRI FOR pENE prediction
Lee et al^68^	Diagnostic and prognostic	Retrospective	134 (105 had both CT and MRI, 17 only CT, 12 only MRI)	HPV related OPSCC	S=62% Sp=77.8% A=71.9% PPV=61.9% NPV=77.8%	rENE did not predict poor PFS in patients with HPV-related OPSCC
Park et al^69^	Systematic review and metanalysis	NA	2478	All HNSCC plus subgroup analysis of HPV+ OPSCC	For CT: Pooled S=73% Sp=83% For MRI: Pooled S=60% Sp=96% CT in HPV+ OPSCC: Sp= 74%	Central node necrosis showed higher sensitivity and infiltration of adjacent planes showed higher specificity.
**Imaging modality- FDG-PET/CT **						
Toya et al^70^	Diagnostic	Retrospective	94	No specific HNSCC subsite	Level-wise analysis S=81.15% Sp=94.35% A=93.1%	SUV_max_ cut-off of 3.0 has diagnostice value in detecting ENE
**Imaging modalities- PET-CT and MRI**						
Sheppard et al^71^	Diagnostic	Retrospective	212 (184: PET-CT 186: MRI and 158 both modalities)	oral cavity, oropharynx, larynx, hypopharynx, lymph node metastasis from squamous cell carcinoma of unknown primary	For PET-CT Significant factors: - clinical stage IV - ill-defined margins - SUVmax > 10 For MRI Marginally significant factors: - ill-defined margins.	Cumulative accuracy 91.43% using the significant features.
**Imaging modality-** CT, MRI, PET-CT						
Abdel-Halim et al^72^	Systematic review and metanalysis	NA	3391	No specific HNSCC subsite	For CT: S=76% Sp=77% For MRI: S=72% Sp=78% For PET-CT: S=80% Sp=83%	PET-CT had significantly higher sensitivity than CT and MRI. Diagnostic ability similar for all.
**Imaging modality-** CT, MRI, PET-CT, US						
Su et al^73^	Systematic review and metanalysis	NA	1155	No specific HNSCC subsite	For CT: S=77% Sp=85% For MRI: S=85% Sp=84% For PET-CT: S=86% Sp=86% For US: S=87% Sp=75%	CT had the lowest sensitivity. CT and MRI had equivalent diagnostic efficacy.

Few researchers have classified ENE on imaging and developed radiological grades of ENE as shown in Table 2.^21^^,^^22^^,^^74–79^ Variability exists regarding the use of necrosis as a criterion for predicting ENE, while some of the researchers, such as Mahajan et al, Aiken et al and Randall et al have found lymph nodal necrosis to be a predictor for ENE, while others have omitted necrosis from their classification.^21^^,^^22^^,^^57^^,^^61^^,^^74–79^ Chin et al, Mao et al, Lu et al and Ai et al graded nasopharyngeal carcinoma (NPC) on MRI. Chin et al, Mao et al and Lu et al included infiltration of tumour into the adjacent structures as grade 3, whereas Ai et al included this category as grade 2.^22^^,^^76^^,^^77^^,^^79^ All three researchers who graded ENE in HPV-positive OPSCC included infiltration of tumour into the adjacent structures as grade/pattern 3.^74^^,^^75^^,^^78^ Mahajan et al classified locally advanced HNSCC on CT and included necrosis as grade 1a and gross muscle/vessel invasion as grade 4.^21^ Coalescent/matted lymph nodes were given grade 2 by Chin et al, Mao et al, Lu et al and Hoebers et al, and labeled pattern 2 by Huang et al, whereas they were not included in the grading by Mahajan et al and Ai et al.^21^^,^^22^^,^^74–79^

**Table 2.** Classifications/grading of extranodal extension on imaging.

Authors						
	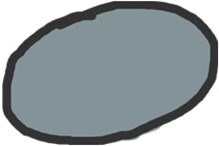 Necrosis	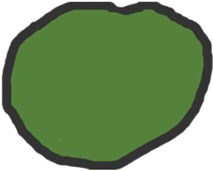 Capsular irregularity	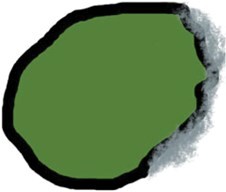 Capsular irregularity with fat stranding	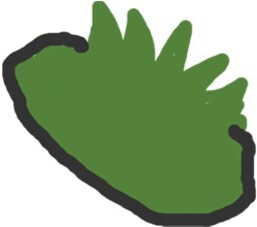 Capsular irregularity with fat invasion through a single node confined to perinodal fat	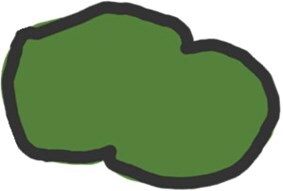 Matted/coalescent nodes	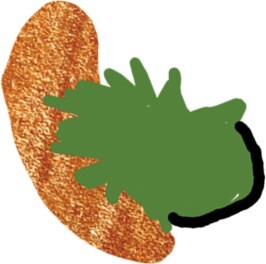 Frank infiltration of adjacent structures (muscles/neurovascular bundles)
Mahajan et al^21^	Grade 1a	Grade 1b	Grade 2	Grade 3		Grade 4
Chin et al^74^				Grade1	Grade 2	Grade 3
Hoebers et al^75^				Grade 1	Grade 2	Grade 3
Chin et al^25^				Grade 1	Grade 2	Grade 3
Mao et al^76^				Grade 1	Grade 2	Grade 3
Huang et al^77^				Pattern 1	Pattern 2	Pattern 3
Lu et al^26^				Grade 1	Grade 2	Grade 3
Ai et al^22^				Grade 1		Grade 2
CT	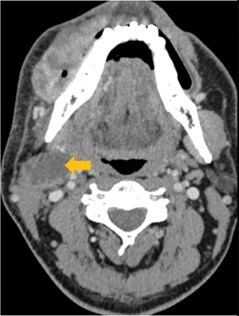	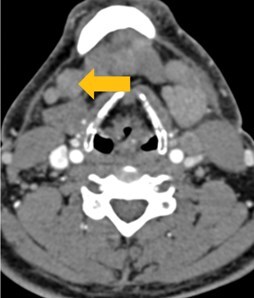	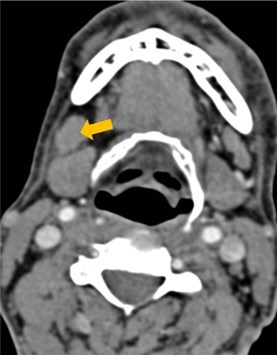	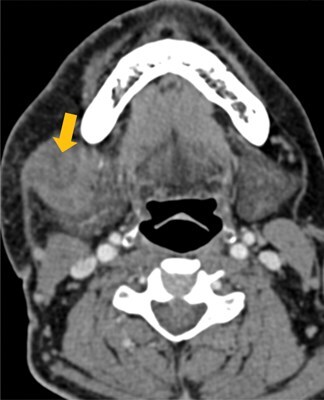	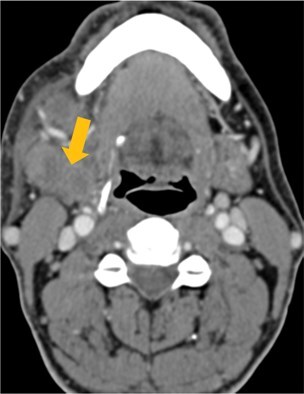	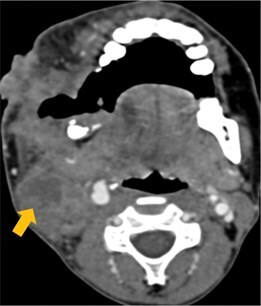
MRI	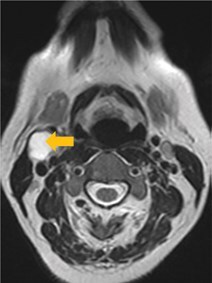	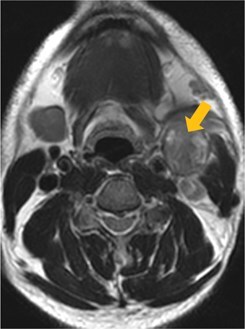	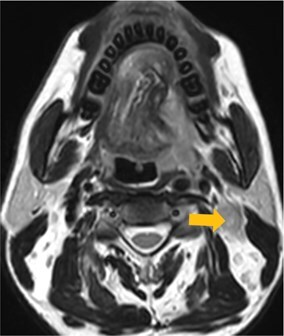	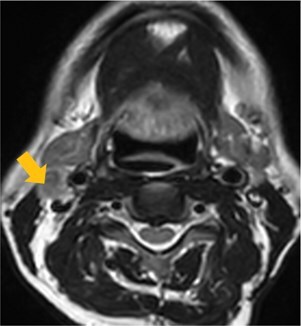	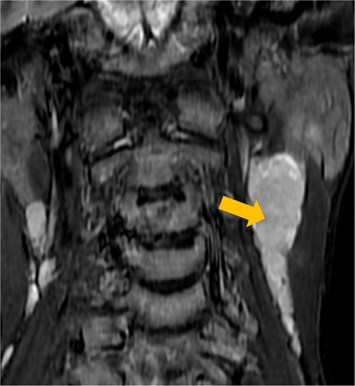	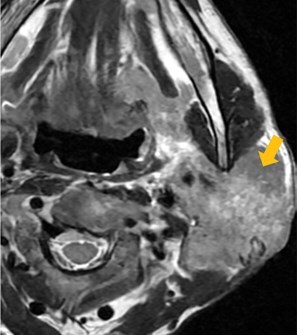
US	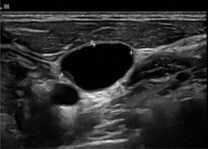	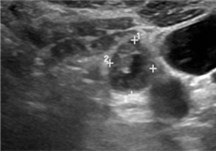	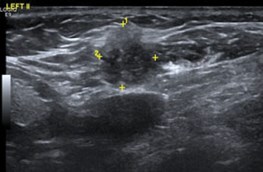	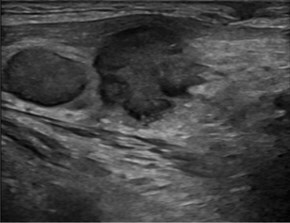	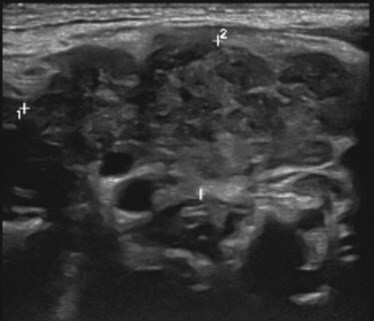	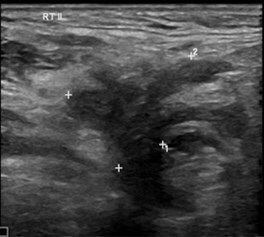

Based on these studies, it is evident that there is a need for standardisation of imaging criteria and a requirement of a consensus on the grading system for rENE.

## Role of ENE in staging

The importance of rENE has further increased with the incorporation of advanced radiological ENE in the N3 category of the latest 9th version of AJCC TNM staging for NPC.^34^ However, rENE is yet to find its place in the staging of the rest of the HNC subsites. Till date, for clinical staging, only unequivocal presence of ENE on physical examination, such as frank involvement of skin, muscle infiltration leading to fixity or tethering of nodal mass, cranial nerve dysfunction, brachial plexopathy, dysfunction of the phrenic nerve or sympathetic trunk, supported by robust radiological evidence, has been suggested to be included as the N3b category in the 8th edition of the TNM staging for cancers of the oral cavity, human papillomavirus (HPV)-negative oropharynx, hypopharynx, larynx and major salivary gland.^80^

Based on the 8th edition TNM staging of HNSCC by the UICC/AJCC for pathological N (pN) category, presence of ENE in a single ≤ 3 cm sized node is given pN2a, whereas ENE in a single node > 3 cm in size or ENE in multiple nodes are assigned pN3b category.^31^

## Role of ENE in treatment planning

Johnson et al in their study, demonstrated that the addition of adjuvant chemotherapy can be tolerated by patients, and providing postoperative adjuvant chemoradiotherapy to patients with ENE in HNSCC may improve their survival.^81^^,^^82^ Simultaneously conducted two randomised landmark trials by the Radiation Therapy Oncology Group (RTOG) and the European Organisation for Research and Treatment of Cancer (EORTC), known as RTOG 9501 and EORTC 22931, respectively, demonstrated that the addition of high-dose cisplatin to radiotherapy benefits postoperative patients having ENE and positive surgical margins in locally advanced HNSCC.^83–85^ The percentages of patients having only ENE in RTOG 9501 and EORTC 22931 were 49% and 41%, respectively.^83–85^ Postoperative adjuvant chemoradiotherapy (CRT) decreased the risk of locoregional relapse (LRR) by 45% in the EORTC trial and 39% in the RTOG trial, and in the pooled analysis, the reduction was 42%.^83–85^ Postoperative adjuvant chemoradiotherapy decreased the risk of treatment failure by 25% in the EORTC trial and 22% in the RTOG trial, and in the pooled analysis, the reduction was 23%.^83–85^

Ten-year follow-up of the RTOG trial revealed a locoregional failure rate of 21% in the chemotherapy plus radiotherapy (RT) group, in comparison to 33.1% for the RT-only group, and disease-free survival (DFS) of 18.4% in the chemotherapy plus RT group, in comparison to 12.3% in the RT-only group.^86^

Based on the findings of these trials, the current management practice is to give adjuvant CRT to those having ENE and/or positive surgical margins.^44^ However, as all the HNSCC are not operated (inoperable OCSCC, laryngeal cancers requiring organ preservation, oropharyngeal and hypopharyngeal cancers which are not operated, and nasopharyngeal cancers in which radiotherapy is the prime treatment modality), increasing reliance is being placed on the role of rENE to prognosticate these patients and plan adjuvant chemoradiotherapy for these patients on baseline imaging itself.

## Implications of rENE in prognostication and clinical outcome

Studies have proven the presence of rENE to be an adverse prognostic factor in HNSCC, as shown in Table 3.^21^^,^^22^^,^^53^^,^^69^^,^^77^^,^^79^^,^^87–90^ Mahajan et al found the rENE-positive group to have a 3-year overall survival (OS) of 46.7% as compared to 63.6% in the rENE-negative group, a DFS of 48.8% in the rENE-positive group as compared to 87% in the rENE-negative group, and a locoregional recurrence free survival (LRRFS) of 39.9% in the rENE-positive group as compared to 60.4% in the rENE-negative group in locally advanced HNSCC.^21^ Almulla et al found 3-year OS of 31% in the rENE-positive group as compared to 68% in those without rENE in OCSCC.^66^ Moon et al showed a 5-year OS and disease-specific survival (DSS) of 74% and 42% respectively in the rENE-positive group as compared to 94% and 84% respectively in rENE-negative group in HNSCC.^27^ Kann et al in their study on OPSCC found that rENE-positive group had a 3-year OS, progression-free survival (PFS) and a distant control of 77%, 71%, and 81%, respectively, which was significantly worse compared to rENE-negative group, however, there was no difference in locoregional control.^28^

**Table 3.** Correlation of radiological ENE with prognosis.

Authors	Nature of study	Sample size	Subsite	Imaging modality	Outcome	Comments
Mahajan et al^21^	Retrospective	354	Locally advanced HNSCC (oropharynx, hypopharynx, and larynx) treated with CCRT	CT	3 year OS: 46.7% LRRFS: 39.9% DFS: 48.8%	rENE status could not be confirmed with pENE
Mao et al^76^	Retrospective	1887	NPC	MRI	5-year OS with grade 3 rENE: N1: 69% N2: 72% N3: 63% 5-year OS without grade 3 rENE: N1: 88% N2: 79% N3: 69%	- Patients with grade 3 rENE in N1 and N2 showed poor outcomes comparable to those in N3. - Based on this RPA generated the following N category: eN0 (N0), eN1 (N1 without grade 3 rENE), eN2 (N2 without grade 3 rENE), and eN3 (N1/N2 with grade 3 rENE and N3)
Karakurt et al^85^	Retrospective	61	NPC	CT and MRI	For rENE+ 5 year OS: 66.7% 5-year DMFS: 70.8% For rENE – 5 year OS: 89.2% 5-year DMFS: 89.2%	rENE is an adverse prognostic factor for poor distant control and OS in patients with NPC.
Chin et al^25^	Retrospective	274	NPC	MRI	For rENE+ OS: 68% DFS: 58% For rENE – OS: 89% DFS: 80%	Proposed unequivocal rENE to be classified to cN3
Lu et al^26^	Retrospective	1616	NPC	MRI	For rENE+ OS: 77.3% 5-year DMFS: 73.8% For rENE – OS: 84.6% 5-year DMFS: 88.4%	- rENE is an independent factor for DMFS and OS but not for LRC. - Proposed N1 grade 2 rENE+ to be reclassified to N2, N2 grade 2 rENE+ to N3, and any N with grade 3 rENE to N3
Ai et al grade 2^22^	Retrospective	546	NPC	MRI	N3 disease using 8th edition AJCC staging: DMFS: 55.6% OS: 54.7% N3 disease using 8th modified edition AJCC staging: DMFS: 51.2% OS: 52.4%	- Stage N1 and N2 nodes with grade 2 ENE were found to have similar DMFS, RRFS, and OS to stage N3 disease. - Proposed grade 2 ENE to be considered as a new criterion for N3 nodal disease in NPC.
Moon et al^27^	Retrospective	117	HNSCC (oropharynx, hypopharynx, and larynx) treated with CCRT	CT	For rENE+ 5-year OS: 74% DSS: 42% CR rate: 66.7% For rENE – 5-year OS: 94% DSS: 84% CR rate: 86.2%	- rENE status were not correlated with pENE. - Pretreatment rENE associated with significantly worse prognosis.
Kann et al^28^	Retrospective	111	Locally advanced OPSCC	CT	For rENE+ 3-year OS: 77% 3-year PFS: 71% 3-year distant control: 81% For rENE – 3-year OS: 95% 3-year PFS: 91% 3-year distant control: 98%	rENE independent adverse prognostic factor for distant control and survival with no significant impact on locoregional control in OPSCC

Abbreviations: CCRT, concurrent chemoradiation therapy; DFS, disease free survival; DMFS, distant metastasis free survival; DSS, disease specific survival; LRC, locoregional control; LRC, locoregional control; LRRFS, locoregional relapse free survival; NPC, nasopharyngeal carcinoma; OPSCC, oropharyngeal squamous cell carcinoma; OS, overall survival; pENE, pathological extranodal extension; PFS, progression free survival; rENE, radiological extranodal extension; RPA, recursive partitioning analysis; RRFS, regional relapse free survival.

## Importance of HPV status on rENE

There is contradictory evidence on the prognostic value of rENE in HPV-positive OPSCC. As shown in Table 1, there have been a few studies to evaluate the diagnostic accuracy of CT/MRI in HPV-positive OPSCC and HNSCC for predicting pENE,^55^^,^^56^^,^^60^^,^^67^^,^^69^^,^^70^ and one of the studies included all the patients of OPSCC, the majority of whom were HPV-positive OPSCC.^62^ Noor et al found CT to be highly specific for predicting pENE.^58^ Faraji et al^53^ found CT features of irregular nodal margins and lack of perinodal fat plane to be the most specific and sensitive for ENE, whereas Geltzeiler et al^64^ showed that the presence of 3 or more suspicious lymph nodes on CT had a 91% positive predictive value (PPV) for the prediction of pENE. Maxwell et al^54^ found that CT could not accurately predict ENE in HPV-positive HNSCC and Patel et al^65^ also showed a low PPV of CT in predicting pENE. Study by Kim et al also showed lower values of sensitivity, specificity, PPV, negative predictive value and accuracy for HPV-positive OPSCC. In the subgroup analysis by Park et al, the pooled specificity of CT in HPV-positive OPSCC was 74%, which was significantly lower as compared to 87% in HPV negative OCSCC, without any statistical difference in sensitivity between the two groups.^69^

As far as prediction of survival outcome is concerned, Lee et al^68^ found that the association between rENE; on CT or MRI, and PFS in HPV-positive OPSCC was not statistically significant, although the trend was towards a worse PFS.

Based on these studies, it can be said that overall imaging is less accurate in predicting pENE in HPV-positive OPSCC as compared to HPV-negative OCSCC, and improved radiological criteria need to be devised for rENE to be clinically applicable in HPV-positive OPSCC.

## Role of rENE in nasopharyngeal carcinoma

The 9th version of the AJCC TNM staging system for NPC now includes advanced rENE in the N3 category, highlighting its clinical relevance, as surgery is not performed in NPC and imaging remains the primary tool for assessing ENE. As shown in Table 3, Karakurt et al, Chin et al, Lu et al and Ai et al have conducted prognostication studies for rENE in NPC and have found reduced survival outcomes in rENE-positive patients.^22^^,^^25^^,^^26^^,^^85^ Karakurt et al found 5-year distant metastasis-free survival and OS of 70.8% and 66.7% in rENE positive group as compared to 89.2% each in rENE-negative group.^85^ Chin et al found that rENE positive group had a lower OS and DFS of 68% and 58%, respectively as compared to 89% and 80%, respectively in rENE-negative group.^25^ Lu et al found that grade2/grade3 rENE had an increased risk of distant metastasis and death, and grade 1 was non-prognostic.^26^ Ai et al found that grade 2 ENE was associated with significantly poorer regional relapse-free survival (RRFS), distant metastases-free survival (DMFS) and OS compared to grades 0 and 1.^22^

## Importance of ENE in papillary thyroid cancer

Various studies have shown that the presence of ENE is an adverse prognostic factor in papillary thyroid cancer (PTC) and is associated with an increased risk of lymph node (LN) recurrence. ENE decreases the possibility of a complete biochemical response after treatment for regional metastatic PTC, enhances the possibility of persistent disease after initial tumour resection, and confers intermediate risk of recurrence when ENE is present in low-volume LN metastasis.^23^^,^^86–88^ A meta-analysis also showed that the presence of ENE conferred a 3.37-fold increased risk of death in patients with PTC.^23^ Qualliotine et al showed that perinodal oedema had the maximum PPV of 83.3% for ENE, and specificity was highest (94.4%) for nodes which were more than 50% cystic.^89^ Although ENE is not part of the current staging system for thyroid cancers, findings from recent studies support its potential inclusion in future staging criteria for PTC.

## Artificial intelligence for predicting rENE

Artificial intelligence (AI) has taken the centre stage in oncologic imaging in recent times, with its ability to automate complex and repetitive tasks, particularly using deep learning (DL).^90^^,^^91^ Increasing evidence and recognition of the role of rENE as a prognostic marker in HNSCC have prompted a few researchers to explore the role of AI in predicting ENE on baseline imaging, as shown in Table 4.^92–95^ Huang et al developed an evolutionary learning (EL) model using radiomics features to predict ENE in HNSCC and achieved an accuracy of 80.00%, a sensitivity of 81.13%, and a specificity of 79.44% for ENE detection on baseline CECT.^92^ Kann et al used a deep learning (DL) model to identify ENE in HPV-positive OPSCC and found superior performance compared to all the four readers.^93^ Kann et al validated a trained deep learning (DL) model on an external institution and The Cancer Imaging Archive (TCIA)- The Cancer Genome Atlas (TCGA) imaging dataset to predict ENE on CT of patients with HNSCC using pathology as the gold standard and achieved an area under the receiver operating characteristic curve (AUC) of 0.84 and 0.90, respectively.^94^ Ariji et al also developed a DL algorithm, and its diagnostic performance was compared to the accuracy of radiologists, and it was observed that the accuracy of DL algorithm was 84%, and that of radiologists based on minor axis ≥ 11 mm, central necrosis, and irregular borders was 55.7%, 51.1%, and 62.6% respectively.^95^

**Table 4.** Artificial intelligence-based studies on the prediction of radiological extranodal extension and their performance status.

Author	Nature of study	Sample size	Imaging modality	HNSCC subsite	AI model	Performance status	Comments
Huang et al^92^	Retrospective	364	CT	No specific subsite of HNSCC	EL model using radiomics	S= 81.13% Sp= 79.44% A= 80%	Features of gray-level texture and 3D morphology of lymph nodes were essential in predicting ENE.
Kann et al^93^	Retrospective	178	CT	HPV+OPSCC	DL model	S= 89% Sp= 72% For ENE identification: AUC: 0.857 For ENE > 1 mm: AUC: 0.859 For nodes with a short-axis diameter of ≥ 10 mm: AUC: 0.74	Performance of DL algorithm for ENE identification was superior to each of the four readers.
Kann et al^94^	Retrospective	82: External institution 62: TCGA imaging data	CT	All subsites of HNSCC including HPV+ OPSCC	Validation of DL algorithm on external institution and TCGA imaging data	For external institution: AUC: 0.84 S= 0.71 Sp= 0.85 A= 83.1% For TCIA-TCGA imaging data: AUC: 0.90 S=0.82 Sp=0.91 A= 88.6% For HPV+ OPSCC (subgroup analysis): AUC: 0.81	AUC of the DL algorithm was superior to both the radiologists for the external data set. For the TCIA-TCGA data set, AUC of DL algorithm was superior to one of the two radiologists.
Ariji et al^95^	Retrospective	51	CT	OSCC	DL	A= 84% Sp= 89.7% S= 66.9%	Small sample size External validation and prospective testing required

Abbreviations: A, accuracy; AUC, area under the receiver operating characteristics curve; DL, deep learning; EL, evolutionary learning; ENE, Extranodal extension; HPV, Human papilloma virus; HNSCC, head and neck squamous cell carcinoma; OPSCC, oropharyngeal squamous cell carcinoma; OSCC, oral cavity squamous cell carcinoma; S, sensitivity; Sp, specificity; TCGA, The Cancer Genome Atlas; TCIA, The Cancer Imaging Archive.

## Conclusion

Radiological ENE, despite its limitations, has emerged as an important means to identify and predict pENE, thereby playing a pivotal role in prognosticating HNC patients and guiding management decisions on baseline imaging itself, especially for those not undergoing surgery. With the inclusion of radiological ENE in the recent 9th version of AJCC staging for nasopharyngeal carcinoma, it is the need of the hour to develop a consensus on the standardised criteria and grading to be used for radiological ENE to enhance its value in rest of the head and neck cancer subsites as well, including HPV-positive HNSCC and papillary thyroid cancers. In addition, radiological criteria for major and minor ENE also need to be developed.
